# Remdesivir for Patients Hospitalized With COVID-19: Evidence of Effectiveness From Cohort Studies in the Omicron Era

**DOI:** 10.1093/cid/ciae515

**Published:** 2024-10-24

**Authors:** Daniel R Kuritzkes

**Affiliations:** Division of Infectious Diseases, Brigham and Women's Hospital, Harvard Medical School, Boston, Massachusetts, USA

**Keywords:** antivirals, real-world evidence, cohort studies, COVID-19, remdesivir

## Abstract

Remdesivir is the only antiviral approved for treatment of persons hospitalized for coronavirus disease 2019 (COVID-19). This supplement presents new information from real-world cohort studies that report reduced mortality in at-risk populations and reduction in readmission for COVID-19 in the Omicron era.

The rapid development of antiviral drugs, monoclonal antibodies, and vaccines to treat and prevent coronavirus disease 2019 (COVID-19) is a notable success in the global response to the severe acute respiratory syndrome coronavirus 2 (SARS-CoV-2) pandemic. Three antiviral drugs, remdesivir, nirmatrelvir/ritonavir, and molnupiravir, have been approved or granted emergency use authorization by the US Food and Drug Administration for the treatment of outpatients with symptomatic COVID-19 at high risk for progression to severe disease. At present, only remdesivir, a nucleotide analog inhibitor of the SARS-CoV-2 RNA-dependent RNA polymerase, is approved for treatment of patients hospitalized for COVID-19.

Conducting randomized, controlled clinical trials of a potentially efficacious investigational drug in the context of a rapidly expanding global pandemic with a high mortality rate proved enormously challenging. A multicenter trial conducted in Wuhan, China, began enrollment in early February 2020, 1 month after public recognition of COVID-19, but closed to accrual just 7 weeks later because public health interventions successfully controlled the spread of SARS-CoV-2 in Wuhan, bringing enrollment in the trial to a halt [1]. With only 237 of an anticipated 423 participants enrolled, the study was underpowered and found no significant difference in time to clinical improvement. The Adaptive COVID-19 Treatment Trial, an international, randomized, double-blind trial of remdesivir vs placebo that enrolled patients hospitalized for COVID-19, demonstrated significant reduction in median time to recovery (10 days vs 15 days, respectively) [2]. Although remdesivir reduced the risk of death by 27%, this effect was not statistically significant [2]. A randomized trial in patients admitted with severe COVID-19 (defined as an oxygen saturation of 94% or less and radiographic evidence of pneumonia) found no significant difference in clinical improvement at day 14 between participants treated with remdesivir for 5 days vs 10 days; because no placebo was included the efficacy of remdesivir could not be determined [3]. A similar trial was conducted in patients hospitalized with moderate COVID-19 pneumonia (presence of pulmonary infiltrates but an oxygen saturation above 94%). However, a placebo arm was included in this trial, and it was found that participants randomized to the 5-day arm had significantly improved clinical status on day 11 compared with placebo; no significant difference was observed for those randomized to the 10-day arm [4]. Whereas initial results of the Solidarity trial, an adaptive platform trial organized by the World Health Organization that compared various interventions to placebo, found no significant benefit of remdesivir [5]; final results showed a significant survival advantage for those randomized to remdesivir among participants who did not were receiving mechanical ventilation at the time of enrollment and showed a significant reduction in progression to ventilation in this group [6].

Given the lack of clarity from these randomized clinical trials, additional data from real-world cohort studies are needed to guide clinicians in their use of remdesivir. A comparative analysis of in-hospital mortality using data from a large multicenter observational cohort showed that patients hospitalized with COVID-19 between August 2020 and November 2020 who received remdesivir had a significantly reduced risk of death at days 14 and 28 [7]. Similar analyses using data collected between December 2020 and April 2022, which included the Delta and early Omicron waves of the pandemic, demonstrated that treatment with remdesivir was associated with reduced 14- and 28-day mortality in various subgroups of hospitalized patients, including those who required and did not require supplemental oxygen and immunocompromised patients [8–10]. Treatment with remdesivir was also associated with a reduced risk of 30-day readmission for COVID-19 [11].

Since those trials and earlier cohort studies were conducted, the nature of the COVID-19 pandemic has changed. Now considered an endemic rather than an epidemic respiratory infection, the severity of COVID-19 appears to have decreased overall due to either lower virulence of the currently circulating Omicron variants or to high levels of immunity that resulted from vaccination or prior infection with SARS-CoV-2. More contemporary data on the effectiveness of remdesivir are therefore needed.

The articles in this supplement provide updated information on the efficacy of remdesivir during the Omicron era in a series of analyses based on data collected through the PINC AI Healthcare Database during the period December 2021 through February 2024. This database captures information from approximately 25% of hospitalizations in 48 states [12]. Results of these analyses confirm and extend to the current era the findings of analyses that used data from earlier phases of the COVID-19 pandemic and demonstrate a persistent benefit of remdesivir treatment for patients hospitalized for COVID-19.

An intrinsic limitation of observational cohort studies is that because treatment is not randomly assigned, observed differences in outcome may be due to differences in patient characteristics rather than to the intervention per se. In the first article in this supplement, Hurwitz et al review propensity score (PS)–based approaches, including inverse probability of treatment weighting (IPTW) and PS matching, for balancing confounding across treatment groups [13]. Although IPTW and PS matching are intended to reduce potential biases by adjusting for known or anticipated confounding factors, these approaches may not fully eliminate the potential for residual confounding. Numerous other factors must be considered when attempting to reduce biases in observational studies, including but not limited to the appropriateness of the data sources, the treatment strategies being compared, the eligibility criteria, and the analytic approach [14]. For these reasons, the results of observational cohort studies must be interpreted with caution and considered together with all available information, including data from randomized clinical trials, when assessing the potential benefits of an intervention in an individual patient.

In the second article, Mozaffari et al use the PINC AI Healthcare Database to evaluate the effectiveness of remdesivir in adults hospitalized for COVID-19 during the Omicron era [15]. This database, formerly the Premier Healthcare Database (www.pinc-ai.com), is a large, geographically diverse, all-payer hospital administrative billing database that is compliant with the Health Insurance Portability and Accountability Act and includes data from approximately one-quarter of hospitalizations in 48 states in the US. After PS matching, 14- and 28-day mortality was compared in 3 groups of patients who did or did not receive remdesivir: adults aged ≥18 years, adults aged ≥65 years, and those with documented COVID-19 pneumonia; all analyses were stratified by oxygen requirement. A statistically significant reduction in the adjusted hazard ratio (aHR) for death of around 20%–25% was observed across all groups and was similar in those who required or did not require supplemental oxygen during the first two days of their hospitalization. A concerning feature of this cohort highlighted by the authors is that 77.8% of the cohort overall received corticosteroids even though 49% were not receiving supplemental oxygen, a group for whom corticosteroid administration may cause harm [16]. Although the reasons for administering corticosteroids to these patients are not known, this observation suggests the need for clinicians to be better educated regarding best practices for patients hospitalized with COVID-19. One limitation of this analysis is that information regarding history of prior COVID-19 or receipt of SARS-CoV-2 vaccination was not provided. In this regard, it is reassuring that the benefits of remdesivir treatment during the late Omicron period (January 2023 through February 2024), when most persons would have had exposure to SARS-CoV-2 either through vaccination or a previous episode of COVID-19, were substantially the same as for the early Omicron period (December 2021 through December 2022).

The third article in this supplement focuses on immunocompromised patients. Because patients with various immunocompromising conditions constituted a relatively small proportion of participants enrolled in the randomized trials of remdesivir or were not analyzed as a prespecified subgroup, information on the efficacy of remdesivir in this important group of patients is lacking from these trials. Given that immunocompromised patients are less likely to mount protective immune responses following vaccination, they remain at high risk for COVID-19 and at greater risk for hospitalization and death if they do acquire SARS-CoV-2 infection. In their analysis, Mozaffari and colleagues analyzed outcomes in nearly 30 000 patients with immunocompromising conditions identified in the PINC AI Healthcare Database who met eligibility criteria [17]. As in the cohort overall, they found that receipt of remdesivir was associated with a significant reduction in the aHR for mortality at days 14 and 28 of 20%–25%. Particularly noteworthy was the finding that treatment with remdesivir lowered the mortality risk at day 28 by 35% in patients with leukemia and by approximately 60% in those with multiple myeloma. A limitation of the analysis is that recipients of solid organ and hematopoietic stem cell transplants were treated as a single group, so that the benefit in these 2 distinct groups of patients cannot be distinguished. In addition, the very small number of patients with human immunodeficiency virus precluded analysis in this particular subgroup.

Given the survival advantage associated with remdesivir treatment demonstrated in the analyses reported above, the observation that nearly 50% of patients hospitalized for COVID-19 in the PINC AI Healthcare cohort did not receive remdesivir, including more than 40% of immunocompromised patients, suggests that a substantial number of deaths could have been averted in 2023 had remdesivir been used more widely in accordance with US guidelines for the treatment of COVID-19. This question is explored in the fourth article in this supplement. Kalil et al explore the potential public health benefits of providing remdesivir to patients hospitalized for COVID-19 in accordance with National Institutes of Health guidelines and emerging evidence-based best practices [18]. They show that if all eligible patients had received remdesivir as recommended, an additional 5.5% of deaths would have been averted. Importantly, a majority of the additional lives saved were among the most vulnerable populations, including the elderly and immunocompromised. As noted by Kalil et al [18], approximately 58 additional persons would need to be treated with remdesivir to avert 1 additional death, a number needed to treat that is commensurate with accepted interventions for influenza, such as use of high-dose quadrivalent vaccine in older patients or treatment with oseltamivir to prevent progression to pneumonia [19, 20].

The final article in this supplement provides evidence that in addition to reducing in-hospital mortality from COVID-19, treatment with remdesivir reduces the risk of readmission for COVID-19 within 30 days of discharge. Mozaffari et al show that treatment with remdesivir was associated with a 22% lower odds of 30-day readmission in the overall study population [21]. This reduction is important not only because of the adverse economic impact of readmission but also because of the high mortality rate (21%) observed in patients readmitted for COVID-19. An important limitation of this analysis is that readmission to a hospital different from the site of initial hospitalization or to a hospital not included in the PINC Database would not have been captured. Similarly, out-of-hospital deaths (though rare) and deaths at hospitals different from the location of initial hospitalization for COVID-19 would not be captured. Thus, the 30-day readmission rates and mortality rates reported in this analysis are likely an underestimate. Assuming that such information is missing equally from treated and untread groups, it is unlikely that underestimation would introduce any significant bias into the analysis.

The strengths of the analyses presented in this supplement include the large number of patients with COVID-19 included in the PINC AI Healthcare Database, the use of both PS matching and IPTW to control for potential confounding, and the consistency of results across subgroups and in various sensitivity analyses. The principal limitations, as with all cohort analyses, are the absence of randomized treatment assignment and potential residual confounding.

In summary, the articles in this supplement provide important new information regarding the efficacy of remdesivir for treatment of patients hospitalized with COVID-19 during the Omicron period. The data presented here confirm and extend the evidence that supports the clinical benefit of remdesivir treatment and provide helpful information for clinicians and guidelines committees.

## Notes

***Financial support.*** This work was supported in part by a grant from the National Institute of Allergy and Infectious Diseases (UM1 AI069412).

***Potential conflicts of interest.*** The author has received honoraria as a consultant to AbbVie, Atea, Decoy, Gilead Sciences, GSK, Janssen, Merck, Moderna, Pfizer, Roche, Shionogi, and ViiV; has provided expert testimony for Janssen and Gilead Sciences, and has received research support from Gilead Sciences, Merck, and ViiV.

***Supplement sponsorship.*** This article appears as part of the supplement “Harnessing Real-world Data to Inform Clinical Practice: Lessons From the Evolving COVID-19 Era,” sponsored by Gilead Sciences, Inc.
